# “Resource Conservation” or “Environmental Friendliness”: How do Urban Clusters Affect Total-Factor Ecological Performance in China?

**DOI:** 10.3390/ijerph17124361

**Published:** 2020-06-18

**Authors:** Peirong Chen, Ruhe Xie, Mingxuan Lu

**Affiliations:** 1 School of Economics and Statistics, Guangzhou University, Guangzhou 510006, China; 1111764008@e.gzhu.edu.cn (P.C.); lumingxuan@e.gzhu.edu.cn (M.L.); 2 College of Economics and Management, Hengyang Normal University, Hengyang 421008, China; 3 School of Management, Guangzhou University, Guangzhou 510006, China; 4 School of Safety and Environment Engineering, Hunan Institute of Technology, Hengyang 421008, China

**Keywords:** urban clusters, resource conservation, environmental friendliness, total-factor ecological performance, extended meta-frontier model

## Abstract

Urban clusters are important carriers for cities to participate in international competition and cooperation, and a booster for urban sustainable development. This study measured the degree of urban clusters by utilizing the panel data of 278 cities in China during 2004–2016. Then, an extended meta-frontier data envelopment analysis (EM-DEA) model was applied to estimate the total-factor ecological performance (UTEP) and decompose it into two sub-index from the perspective of “resource conservation” and “environmental friendliness”. On these bases, we employed a dynamic panel data approach to examine and demonstrate the relationship between urban cluster and UTEP in two dimensions, and further explored transmission channels of urban clusters on UTEP by adding the mediating effect. The results show that resource conservation increases first and then decreases with the increasing of urban clustering level, while environmental friendliness showed the opposite trend, making the latter become the main way for urban clusters to improve the UTEP. Industrial structure supererogation and rationalization are transmission channels for environmental friendliness rather than resource conservation in the way of improvement of UTEP. Technology innovation, as well as technology diffusion, also improves UTEP to some extent. In addition, urban clusters in eastern and central China have the greatest improvement in UTEP, while such effects are not the case in western China. Urban clusters in the second half sample period are more conducive to the improvement of the UTEP, with these potentially being the gains from the improvement of the level and quality of urban clusters.

## 1. Introduction

In recent decades, China’s urbanization rate has increased from 17.92% to 59.58% between the years 1978 and 2018. As a large number of people have been moved from rural areas to cities, the number and size of cities in China have greatly expanded. Although the rapid expansion of urbanization provides a driving force for the rapid development of China’s economy, it has caused increasing pressures on resource conservation and environmental protection [1,2,3]. Up until now, there are two different paths towards China’s urbanization. One is “big urbanization”, which completes the urbanization process by encouraging the development of metropolises and megacities [4]. The other is “small urbanization”, which focuses on the development of small cities and finishing the urbanization process through high-quality development of small cities and towns [5]. The former emphasizes the efficiency of “intensive” urbanization, while the latter emphasizes the low cost of “decentralized” urbanization. The development of urban clusters can not only exhibit the high-efficiency characteristics of “intensive” urbanization, but also can take into account the coordinated development of megacities and small/medium-sized cities. Urban clusters are geographic concentrations of urban places located within a communicable range of each other. An “urban cluster” includes major cities or, alternatively, is shaped by urban settlements of similar size; urban agglomerations are a specific form of urban clusters.

Ambitious Chinese policymakers are planning a new blueprint for the development of urban clusters. Compared with other countries, China’s urban cluster construction has the following characteristics. First, the scale of the urban cluster is huge. Although the Greater Tokyo area is currently the world’s biggest urban cluster, with a population of around 40 million, the population of the Yangtze River Delta urban agglomeration is almost four times that of Greater Tokyo [6]. Second, China’s urban clusters have built an efficient and connected urban network through high-speed rail networks, especially inter-city high-speed railways, and have expanded the coverage of urban clusters. Third, with the top-down nature of urban clusters, China can implement stronger government intervention.

Urban clusters have become the place with the greatest potential and vitality for economic development in China. At the same time, they are also the place where eco-environmental problems are most concentrated. Many academicians and experts in urban economics pointed out at the “China City 100 Forum” in 2014 that China’s urban clusters have become “pollution clusters”—the pollution levels of the three major urban clusters are significantly higher than the national average. Recent years have seen increasing regional planning and development policies targeting sustainable development of urban agglomerations. In December 2007, the China State Council approved the “Wuhan urban group” and “Chang-Zhu-Tan urban agglomeration” as “national comprehensive reform pilot areas for resource-saving and environment-friendly society construction”, which provide a clear ecological road for the development of China’s urban clusters. In 2012, the “12th Five-Year Plan for the Prevention and Control of Air Pollution in Key Regions” was issued, and it was proposed that a regional air pollution joint prevention and control mechanism in key urban agglomerations would be established. In particular, the “National New Urbanization Plan (2014–2020)” emphasizes “the development of urban clusters with high cluster efficiency and strong functional complementarities”. Urban clusters have had a profound impact on regional resource utilization and environmental protection. Therefore, how can urban clusters affect the regional ecological environment while achieving economic development? Will the impact change with the different conditions of economic and social development? These are issues of interest to many urbanists and economists.

China has been undergoing the largest and fastest construction of urban clusters around the world. The sustainable development of urban clusters needs to consider the carrying capacity of the ecological environment, that is, to achieve resource conservation and environmental improvement as much as possible while developing the economy. The concept of ecological performance was first put forward in 1990 and has been endowed with new connotations by World Business Council for Sustainable Development (WBCSD). According to the WBCSD’s redefinition, products and services can also decrease ecological impacts and resource consumption to a level that matches the carrying capacity of the earth in relation to life cycle [7]. Therefore, in this paper, urban total-factor ecological performance is the measure of economic output used with respect to resource input and ecological environment cost in the cities. Data envelopment analysis (DEA) has been widely used to measure the efficiency of resources and the environment, and researchers are further promoting the expansion and application of this method [8,9]. However, to measure the total-factor ecological performance more accurately, we need to consider several issues such as technical heterogeneity [10], slack-based measure [11], and the problem of “technical regress” [12].

The ecological environment problems arising from the construction of urban cluster need to be solved in two ways: source control and end-of-pipe control [13]. In the economy, this corresponds to “resource conversation” and “environmental friendliness”. Therefore, it is of great significance to study the impact of urban clusters on total factor ecological performance from the two dimensions of resource conservation and environmental friendliness. Methodologically, the index decomposition method based on DEA can achieve the organic unity of the total-factor ecological performance and its decomposition index under the endogenous conditions of resource consumption and pollution discharge. For example, Chung et al. [14] used the Malmquist-Luenberger index to decompose total factor productivity (TFP) into efficiency change and technological change to study the growth of green TFP. However, the decomposition index calculated is still a total evaluation index integrating all inputs and outputs, and the total factor ecological performance cannot be evaluated from the two dimensions of resource consumption and pollutant discharge. Therefore, from the perspective of input and output, this paper will decompose total-factor ecological performance into two sub-indexes under the DEA framework from the two dimensions of “resource conservation” and “environmental friendliness”.

On the basis of the analysis mentioned above, we contributed to this article in the following ways. First, we developed an extended meta-frontier DEA (EM-DEA) method to estimate the urban total-factor ecological performance (UTEP) more accurately. The EM-DEA considers the technical heterogeneity caused by regional differences when constructing the technological frontier function. Meanwhile, this method overcomes the problem of horizontal incomparability when multiple efficiency values are 1, and the problem of vertical incomparability caused by technological progress when using panel data. Second, starting from the source control (resource conservation) and end-of-pipe control (environmental friendliness) of eco-environmental problems, we decomposed UTEP into urban total-factor resource performance (UTRESP) and urban total-factor environmental performance (UTENVP) under the unified measurement framework of DEA. On this basis, we established a theoretical analysis framework to analyze how urban clusters affect the UTEP from the two dimensions of resource conservation and environmental friendliness. Finally, we employed the mediating effect model to identify the ways and effects of urban clusters affecting UTEP, and also focused on the difference in the mediating effects of supererogation and rationalization of the industrial structure in the two dimensions of resource conservation and environmental friendliness.

The rest of this article is structured as follows. Section 2 introduces the literature review and proposes research hypothesis. Section 3 introduces the measuring approach and econometric model. Section 4 supplies data description and variable definitions. Section 5 presents the basic estimated results and discussion. Section 6 reports the results of the heterogeneity analysis. Section 7 provides conclusions and relevant policy recommendations.

## 2. Literature Review and Research Hypothesis 

### 2.1. Literature Review

#### 2.1.1. The Economic Effect of Urban Clusters

The rapid development of urban clusters has attracted widespread attention from scholars, and there has been literature discussing the economic effects of urban clusters, such as the growth effect [15,16], productivity improvement effect [17,18], and increasing employment opportunities effect [19]. Only a small amount of the literature has discussed the relationship between urban clusters and regional green development under the constraints of the ecological environment, with the related literature mainly focusing on the following two categories. One focuses on comparing the differences in the level of ecological development among special urban clusters. For instance, Tao et al. [20] compared the green total factor productivity of China’s three major urban agglomerations and found that the cumulative growth rate of the Yangtze River Delta urban agglomerations is higher than the Beijing–Tianjin–Hebei urban agglomerations and the Pearl River Delta urban agglomerations. Tang and Li [21] discussed in detail the reasons for the significant differences in the level of green development among the three major urban agglomerations. The other is about the relationship between the spatial structure of urban clusters and regional ecological development. According to Borck and Pflüger [22], the spatial structural asymmetry between central cities and peripheral cities will increase overall emissions. Xie et al. [23] believe that the land efficiency of the provincial capital cities in the urban agglomerations is significantly higher than that of the surrounding cities. Song et al. [24] pointed out that the existence of spatial dependence is one of the main characteristics of urban clusters, and that the increases in the intensity of urban spatial networks reduce the intensity of regional CO_2_ emissions. The aforementioned literature explored the influencing factors of urban ecological environmental changes from various aspects, paying particular attention to the role of spatial factors in the evolution of the ecological environment, including some scattered research from the perspective of urban clusters. For example, Huang et al. [25] compared the ecological efficiency of urban agglomeration and non-urban agglomeration in the Yangtze River Economic Belt and found that the former was significantly higher than the latter. However, these studies only studied the ecological development level of specific urban agglomerations and their comparison. One of the most relevant studies in the literature gives a general answer to the impact of urban clusters on ecological performance [26], but does not answer whether the impact is due to resource conservation or environmental friendliness. Moreover, the influence mechanism of urban clusters on them also needs to be further expanded. Therefore, there is still a large space to systematically explore the relationship between urban clusters and ecological performance.

#### 2.1.2. Related Research on Ecological Performance

The existing literature has carried out a series of studies on the concepts, measurement methods, and measurement indicators related to urban total-factor ecological performance. Total-factor ecological performance, which simultaneously takes economic growth, resource consumption, and environmental cost into consideration, is an effective tool to evaluate the comprehensive development level of regions [27]. Data envelopment analysis (DEA) is widely used in effectively evaluating ecological performance, which is the basis for further exploring its relationship with urban clusters [28,29]. The general DEA method is based on the assumption of homogeneity; that is, each decision-making unit (DMU) has the same technological frontier. However, China has a vast territory, and cities in different regions have huge difference in ecological environment, institutional background, and management capabilities. Therefore, the regional heterogeneity must be considered when constructing the technological frontier, otherwise the evaluation results will be biased [30]. O’Donnell et al. [31] proposed the concept of meta-frontier to divide DMUs with different production technologies into groups, and using the technology gap ratio (TGR) to represent the heterogeneity between group technology and meta-frontier technology. Zhang and Choi [32] combined the meta-frontier model with the non-radial directional distance function (NDDF) to complete the measurement of carbon emission performance. However, the above methods often obtain a TGR greater than 1, which contradicts the assumption that TGR must be between 0 and 1. Wang et al. [8] proposed a modified meta-frontier method, effectively avoiding the situation that TGR exceeds 1. In addition, ecological performance is a comprehensive indicator that considers all inputs and outputs (including desirable and undesirable outputs). Most empirical research involves analysis from the perspective of total performance value, and does not accurately describe the internal logic between total performance and sub-factor performance. Zhou et al. [33] regard the weighted average of energy input and carbon output as the total carbon emission performance, providing inspiration for the present paper to explore the relationship between urban clusters and ecological performance from the two dimensions of resource conservation and environmental friendliness.

In terms of the selection of inputs and outputs, the existing research has great flexibility in the selection of resource and environmental indicators. Wursthorn et al. [34] established the accounting framework of ecological efficiency in European countries on the basis of economic and ecological indicators. Li et al. [35] developed a comprehensive method to measure eco-efficiency in the manufacturing process by considering energy efficiency and resource efficiency. Cheng et al. [28] measured the ecological efficiency of China’s Yangtze River Economic Belt by taking energy and water as resource indicators and wastewater, sulfur dioxide, soot, and dust as environmental indicators. In addition, scholars considered a broader resource conception in the input–output indicators, such as construction land [27]. Adjei Kwakwa et al. [36] assessed the eco-system on the basis of the relationship between natural resource development and carbon dioxide emissions. The research on the indicator selection mentioned above provides solid theoretical underpinnings for the construction of the inputs and outputs. This article will determine the corresponding indicators on the basis of the concept of ecological performance, existing research, and data availability.

### 2.2. Research Hypothesis

The development of urban clusters has gathered a large number of economic production activities and has become a new growth point of China’s economy. At the same time, China’s cities are facing unprecedented ecological environment pressure due to the huge consumption of natural resources and the emission of pollutants [37]. Therefore, improving the performance of resource conservation and emission reduction in urban clusters is of great significance to China’s sustainable development. For example, the National New Urbanization Plan (2014–2020) emphasizes that China’s three major urban clusters should play an important role in China’s energy conservation and emission reduction tasks. The achievement of the above goals is basically determined by source control (resource conservation) and end-of-pipe control (environmental friendliness)—improving resource efficiency and reducing pollution intensity. Therefore, this article studies the relationship between urban clusters and urban total-factor ecological performance from the two dimensions of resource conservation and environmental friendliness.

#### 2.2.1. Resource Conservation or Environmental Friendliness?

As a type of spatial agglomeration in a larger scope, urban cluster is an important organizational form of economic space activities. On the one hand, compared to decentralized production, concentrated production can bring advantages such as economies of scale and increasing returns to scale [38]. With the improvement of concentration levels, urban clusters can effectively save production costs of enterprises, promote the centralized utilization of resources, and reduce waste of resources [26,39]. In addition, urban clusters are conducive to breaking market segmentation by strengthening the economic and technological links between cities [19], which helps each city to carry out industrial division and cooperation on the basis of their own resource endowment and competitive advantages, as well as optimizing the rational allocation of resources. Increasing the degree of clustering will also form network externalities and thus exert effects on the whole region covered by the urban cluster system [40]. These effects increase productivity while reducing resource consumption, and ultimately contribute to resource conservation. On the other hand, urban clustering, along with the expansion of production scale and the increase of input factors, consumes a large amount of energy, land, water, and other resources. In particular, China’s big cities (provincial capitals) have unique advantages in politics and economy, and they have a huge siphon capacity to the surrounding cities [41], which may make it easier for urban economic activities to exceed the reasonable capacity, causing a congestion effect and thereby causing vicious competition between enterprises. On the contrary, it offsets some or all of the “scale effect” and has a negative impact on urban sustainable development. China in particular is currently in the stage of rapid urbanization and industrialization, and the consumption of various resources continues to climb with strong rigidity. Thus, in light of the argument above, we proposed the following hypothesis:

**Hypothesis 1a** **(H1a).** *The impact of urban clusters on resource conservation depends on the relative strength of scale effect, network effect, congestion effect, and competition effect. When the scale effect and network effect exceed the congestion effect and vicious competition, urban clusters promote resource conservation, and vice versa*.

Generally, the relationship between economic development and environmental pollution can be described as the “environmental Kuznets curve” [42]. In earlier times, the rapid development of urban clusters is accompanied by a large number of production activities concentrated in cities, especially the concentration of a large number of heavily polluting industries, which has a negative impact on environmental quality while developing the economy [43,44]. For example, some studies showed that industrial pollutants had become the main source of pollutant emissions, and industrial agglomerations have increased emissions of multiple pollutants, including waste gas, wastewater, and waste residue [45,46]. At the same time, there is a two-way mechanism between economic agglomerations and environmental pollution-economic agglomerations aggravate environmental pollution, and environmental pollution has a negative inhibition effect on economic agglomerations [47], while urban clustering can prevent excessive concentration of economic activity in a single city. In addition, when urban clusters are in the relatively mature stage, it will exhibit various positive externalities and have a positive effect on the improvement of the environmental quality. For example, Zhu and Xia [37] pointed out that accumulated industrial spatial clusters will increase pollution emissions first and then reduce pollutant emissions with the increase of urbanization level. Lu and Feng [48] showed that a reasonable concentration of population and economic activity is conducive to reducing the intensity of industrial pollutant emissions. The possible reasons are (1) clusters effectively reduce commuting distances and improve environmental quality; thus, clustered cities are more environmentally friendly than decentralized villages [49]. (2) Clusters are conducive to technological innovation and diffusion [50], especially the continuous development of clean technology, reducing emission levels, and improving environmental quality. (3) The development and maturity of urban clusters have strengthened the links between cities, facilitated the exchange and cooperation between central cities and peripheral cities, and ultimately helped to solve the diseconomies of scale in big cities. Thus, we propose hypothesis 1b:

**Hypothesis 1b** **(H1b).** *The impact of urban clusters on environmental friendliness depends on its stage of development. When the urban clusters are in the stage of development, the speed of environmental deterioration exceeds the speed of economic expansion, and urban clustering suppresses the environmental friendliness. When urban clusters are in the mature stage, the speed of economic expansion is faster than the speed of environmental deterioration, and therein the urban cluster promotes environmental friendliness*.

#### 2.2.2. Transmission Channels for Urban Clusters on Regional Ecological Performance

The forming process of urban clusters is a process of interaction between cities, and the flow of production factors is a manifestation of spatial interaction between cities. The flows of capital, technology, and talents, and the areal division of labor determine the evolution of the spatial structure and industrial structure in an urban cluster [51]. Cities in the clusters rely on their own endowments, characteristics, and advantages, undertaking different functional division, and thus the urban clusters have comprehensive region functions, industrial cooperation advantages, and structural effects [25]. The closer the industrial collaboration between cities, the more the urban clusters can work together instead of disorderly competition of individual cities, thus reducing waste of resources and obtaining the adjustment effect of industrial structure [52]. Ignoring their interaction will separate the role of urban network and industrial structure adjustment in resource saving and pollution reduction.

The industrial structure adjustment of urban clusters is mainly through the following two approaches. The first is the supererogation of industrial structure. When the industrial structure is upgraded from the secondary industry to the tertiary industry, regional pollution levels will be reduced to a certain extent [53]. Urban clustering strengthen the advantages of the central city, which can help the central city to eliminate the backward industries with high pollution, and attract clean and high-end industries, especially the tertiary industry. The second is the rationalization of the industrial structure. The flow of production factors from low-efficiency sectors (enterprises) to high-efficiency sectors (enterprises) helps to balance the productivity between industries, thereby realizing the rationalization of the industrial structure. In particular, when excessive economic activities are absorbed and cause “urban congestion”, the close economic and technological ties between cities in the urban clusters help the central city to transfer some industries to peripheral cities with comparative advantages. In one path, it prevents duplicate construction and alleviates the “big city disease”. In another, other cities within an urban cluster can undertake competitive production functions on the basis of their own advantages, optimize the input–output structure, and improve ecological efficiency. Thus, we propose the following hypothesis 2a:

**Hypothesis 2a** **(H2a).** *The urban clusters accelerate the supererogation and rationalization of industrial structure, which may help to achieve the resource conservation and environmental friendliness effects*.

There is evidence that urban clustering could stimulate innovation [19,54]. The spatial agglomeration externalities and network externalities are important ways for urban clusters to generate technological innovation and diffusion [55]. Firstly, under the government planning and market mechanisms, central cities are more likely to attract high-quality production factors such as talents, technology, and information, and then form a relatively independent and concentrated cluster economy with endogenous development capabilities. Geographical proximity reduces the cost of communication between companies in a cluster economy and facilitates the exchange of face-to-face invisible knowledge (an important driver of innovation) [56], which enables sharing, matching, and learning mechanisms, and provides conditions for the development and application of new production technologies, energy conservation, and emission reduction technologies. This is called agglomeration externality. Secondly, a growing body of evidence shows that although knowledge exchange and spillover mainly depend on the local network, extra-local connections are particularly important to innovation [57], and urban clusters will facilitate the exchange of knowledge on a larger range region (within and between cities), with these cluster effects being called network externality. Producer services such as scientific research, accounting affairs, consulting information, and financial services are suitable for diversified agglomeration in regional central cities with large market scale [58]. In one path, industrial integration is conducive to the formation of emerging industries in urban agglomerations; in another, significant economic externalities are generated on surrounding cities by expanding the service radius of central cities. For example, Yu et al. [59] used spatial econometric analysis and found that the technological progress of a city affected not only local carbon emissions but also that of neighboring cities. Finally, with the improvement of the level of urban clusters, central cities are more closely connected with peripheral cities, forming a social production network that divides labor and leads to cooperation, accelerating the spatial concentration and diffusion of production factors such as technology, talents, and information, all of which help peripheral cities to improve productivity, save resources, and reduce emissions. Bertinelli and Black [60] pointed out that with the accumulation and overflow of knowledge capital in a single city, the urban agglomeration formed after the number of cities increases has higher economic efficiency. Thus, we propose the following hypothesis 2b:

**Hypothesis 2b** **(H2b).** *Urban clusters may generate resource conservation or environmental friendliness through technology innovation and diffusion*.

**Figure 1:**
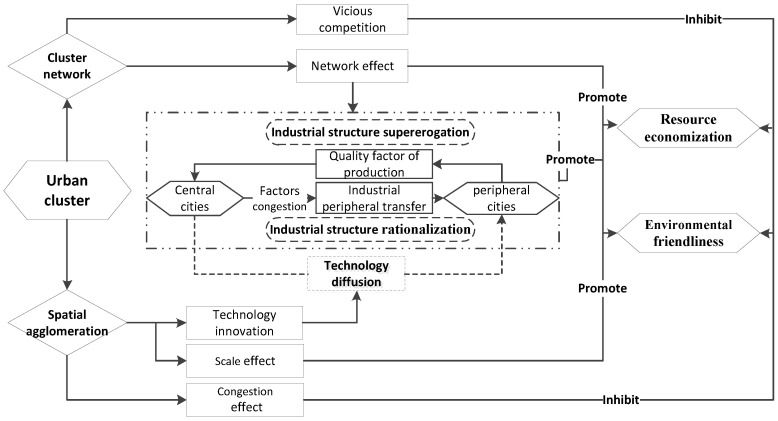
Diagram of analytical framework.

On the basis of the arguments above, an analytical framework is drawn in Figure 1. Urban clusters exhibit the dual characteristics of cluster networks and spatial agglomeration, and have an impact on total-factor ecological performance from two dimensions of resource conservation and environmental friendliness. On the one hand, a healthy urban cluster can make full use of the advantages of various cities in terms of resource endowment and industrial characteristics, forming a benign industrial cooperation network and optimizing resource allocation. The exchange and cooperation between the central city and the peripheral cities, while driving the economic development of the peripheral cities, also relieves the uneconomical scale of the central cities. At the same time, vicious competition is also a concern for the rapid expansion of urban agglomerations. On the other hand, the scale effect of spatial agglomeration can directly improve the resource utilization effect and pollutant emission efficiency. However, excessive spatial agglomeration will produce a crowding effect, which is not conducive to the conservation of resource consumption and the improvement of environmental quality. These are what hypotheses 1a and 1b mentioned. In addition, technological innovations generated by urban clusters, especially green technologies, can achieve technology diffusion through the use of urban networks, and can play a role in improving the resource consumption and environmental quality of technological externalities. Meanwhile, the optimization of industrial collaboration networks is another path. The central cities attract a large number of high-quality production factors, such as talents, which contribute to the supererogation of the industrial structure, while the peripheral cities undertake industrial transfer according to their advantages to realize the rationalization of the industrial structure. These are mentioned in hypotheses 2a and 2b. 

## 3. Methodology

### 3.1. EM-DEA Model for Measuring UTEP and Its Decomposition Index

In this paper, the UTEP is decomposed into total-factor resource performance (UTRESP) and total-factor environmental performance (UTENVP) under a unified measurement framework in the extended meta-frontier model, so as to distinguish the resource conservation and environmental friendliness effects of urban clusters. The construction of the production possibility set is the premise of analyzing efficiency or performance. Suppose there are i = 1,…, R cities, t = 1,…,T periods; each city is regarded as a decision-making unit (DMU). Production technologies in different regions are affected by the economic, geographical, market, or other factors. Therefore, all DMUs need to be divided into H subgroups when constructing their technological frontiers. The number of cities in the h-th group is $N^{h}=R_{h}^{1}\cup R_{h}^{2}\cup \cdots \cup R_{h}^{T}$, and $\sum_{h=1}^{H}N^{h}=N$. Each group has its own specific production technology, and the DMUs in the same group have the same frontier of group technology.

Assuming that each DMU produces M desirable outputs, $y=[y^{1},y^{2},\ldots y^{M}]\in R_{+}^{M}$, and S undesirable outputs, $e=[e^{1},e^{2},\ldots e^{S}]\in R_{+}^{S}$, by using K inputs, $r=[r^{1},r^{2},\ldots r^{K}]\in R_{+}^{K}$. $P^{h}$ and $P^{m}$ are the production technology sets under the group-frontier and meta-frontier, respectively. According to O’Donnell et al. [31], we define $P^{m}=\left\{P^{1}\cup P^{2}\cup \cdots \cup P^{H}\right\}$. Yu et al. [30] pointed out that different cities in eastern, central, and western China used different types of production technologies due to their variations in resources endowment, governance capability, and development stage of economy. In other words, the technology heterogeneity existed among the three regions in China. Therefore, we divided all samples into three groups by region to measure the UTEP of group-frontier. On the basis of the above assumptions, this paper constructed the production technology set of group h-th under the assumption of constant returns to scale (CRS) as follows.

(1) $$P^{h}\left\{(r^{h},y^{h},e^{h}):\sum_{i=1}^{N_{h}}\lambda_{i}^{h}r_{i}^{h}\leq r^{h};\sum_{i=1}^{N_{h}}\lambda_{i}^{h}y_{i}^{h}\geq y^{h};\sum_{i=1}^{N_{h}}\lambda_{i}^{h}e_{i}^{h}=e^{h};\lambda_{i}^{h}\geq 0,i=1,2,\cdots ,N^{h}\right\}$$

where $\lambda_{i}^{h}$ is intensity variable for constructing the production technology into a convex expression, and $P^{h}$ satisfies the hypothesis of weak disposability of inputs, desirable outputs, and undesirable outputs. Following Beltrán-Esteve et al. [29], we then defined the non-radial directional distance function as follows:

(2) $${\vec{D}}^{h}(r,y,e;g^{h})=\sup\left\{w^{T}\beta :((r,y,e)+g\cdot diag(\beta ))\in P^{h}\right\}$$

where $w={\left(w_{r},w_{y},w_{e}\right)}^{T}$ is the normalized weights vector corresponding to the input–output variables, $g=(-g_{r},g_{y},-g_{e})$ is the direction vector, and $\beta ={\left(\beta_{r},\beta_{y},\beta_{e}\right)}^{T}\geq 0$ represents the proportion of each input–output variable that can be expanded or reduced. To overcome the “discriminating power problem” of multiple DMUs [61,62], Tone [11] proposed the non-radial super-efficiency model. To ensure the accuracy of the calculation, the extended meta-frontier model combined the idea of super efficiency and group benchmark technology, which can be expressed as follows:

(3) $$\begin{array}{l} \vec{D^{h}}(r,y,e;g^{h})=\max\{w_{r}\beta_{r}^{h}+w_{y}\beta_{y}^{h}+w_{e}\beta_{e}^{h}\}\\ s.t.\;\sum_{i=1,i\neq o}^{N_{h}}\lambda_{i}r_{i}^{k}\leq (1-\beta_{r}^{h})r_{o}^{k},\forall k\\ \;\sum_{i=1,i\neq o}^{N_{h}}\lambda_{i}y_{i}^{m}\geq (1+\beta_{y}^{h})y_{o}^{m},\forall m\\ \;\sum_{i=1,i\neq o}^{N_{h}}\lambda_{i}e_{i}^{s}=(1-\beta_{e}^{h})e_{o}^{s},\forall s\\ \;\lambda_{i}\geq 0;\beta_{r}^{h},\beta_{y}^{h},\beta_{e}^{h}\geq 0;p=1,\cdots ,N^{h};h=1,2,\cdots ,H \end{array}$$

The specific input variables in this article include capital (K), labor (L), energy (E), land resources (B), and water resources (W); the desirable output is urban GDP (U), and the undesirable outputs are wastewater (D) and waste gas (S). Considering the alternatives between input factors, we stripped the inefficiencies of K and L to maximize the potentiality of natural resource consumption and pollution emissions [63,64]. In addition, we assumed that the inputs, desirable outputs, and undesirable outputs were equally important. Therefore, the weights vector was set to $w^{T}={\left(0,0,w_{E}^{},w_{B}^{},w_{W}^{},w_{U}^{},w_{D}^{},w_{S}^{}\right)}^{T}=$${\left(0,0,1/9,1/9,1/9,1/3,1/6,1/6\right)}^{T}$. The direction vector corresponding to the input–output variables is $g=(0,0,-x_{hp}^{E},-x_{hp}^{B},-x_{hp}^{W},y_{hp}^{U},-b_{hp}^{D},-b_{hp}^{S})$. If $\vec{D^{h}}(r,y,e;g^{h})=0$, the evaluated DMUs are at the best practice frontier, which is efficient in the direction of the vector. Once Equation (3) is solved, we can easily obtain the optimal solutions $\beta^{h*}={\left(\beta_{E}^{h*},\beta_{B}^{h*},\beta_{W}^{h*},\beta_{U}^{h*},\beta_{D}^{h*},\beta_{S}^{h*}\right)}^{T}$ on the basis of the group-frontier. If $\beta_{z,hi}^{h*}=0(z=E,B,W,U,D,S)$, the decision-making unit achieves the optimal value on the inputs (or outputs).

Similar to Equation (3), the distance function of the oth DMU in group p ($o=1,2,\cdots ,N_{P};$ p=1,2,⋯,H) under the meta-frontier can be estimated by the following Equation (4), where, $\left(1-\beta_{r}^{h*}),(1+\beta_{y}^{h*}\right)$ and $\left(1-\beta_{e}^{h*}\right)$ are obtained by solving Equation (3) before estimating Equation (4). Variables $\beta_{r}^{m}$,$\beta_{y}^{m}$ and $\beta_{e}^{m}$ are added to project each DMU under the meta-frontier. Once Equation (4) is solved, we can easily obtain the optimal solutions $\left(1-\beta_{r}^{m*}\right)$, $\left(1+\beta_{y}^{m*}\right)$ and $\left(1-\beta_{e}^{m*}\right)$, which represent the improvement space from group-frontier to meta-frontier. In other words, $\left(1-\beta_{r}^{\;m*}\right)$, $\left(1+\beta_{y}^{m\;*}\right)$ and $\left(1-\beta_{e}^{m*}\right)$ can measure the technology gap between group-frontier and meta-frontier. The reason why we used this method was that the direction of the directional vector $g^{h}$ under the group-frontier did not equal to the direction of the directional vector $g^{m}$ under the meta-frontier when we used the non-radial directional distance functions for measuring ecological performance. As such, the technology gap ratio (TGR) may exceed unity in some situations. However, if $\beta_{r}^{m*}$, $\beta_{y}^{m*}$, and $\beta_{e}^{m*}$ obtained by this method are not less than zero, we can be assured that the TGR is not greater than unity [8], and thus the performance value of meta-frontier will not exceed that of group-frontier. This is the difference from the traditional meta-frontier that uses radial directional distance functions for measuring technology gap and ecological performance.

(4) $$\begin{array}{l} \vec{D^{m}}(r,y,e;g^{m})=\max\{w_{r}\beta_{r}^{m}+w_{y}\beta_{y}^{m}+w_{e}\beta_{e}^{m}\}\\ s.t.\;\sum_{h=1}^{H}\sum_{i=1(i\neq o\;if\;h=p)}^{N_{h}}\lambda_{hi}r_{hi}^{k}\leq (1-\beta_{r}^{m})(1-\beta_{r}^{h*})r_{po}^{k},\forall k\\ \;\sum_{h=1}^{H}\sum_{i=1(i\neq o\;if\;h=p)}^{N_{h}}\lambda_{hi}y_{hi}^{m}\geq (1+\beta_{y}^{m})(1+\beta_{y}^{h*})y_{po}^{m},\forall m\\ \;\sum_{h=1}^{H}\sum_{t=1(i\neq o\;if\;h=p)}^{N_{h}}\lambda_{hi}e_{hi}^{s}=(1-\beta_{e}^{m})(1-\beta_{e}^{h*})e_{hp}^{s},\forall s\\ \;\lambda_{hi}\geq 0;\beta_{r}^{m},\beta_{y}^{m},\beta_{e}^{m}\geq 0;h=1,\cdots ,H;i=1,\cdots ,N^{h};h=1,2,\cdots H\; \end{array}$$

Solving Equation (4), the target value of inputs, desirable outputs, and undesirable outputs are equal to $(1-\beta_{k,hi}^{m*})(1-\beta_{k,hi}^{h*})r_{hi}^{k}$, $(1+\beta_{m,hi}^{m*})(1+\beta_{m,hi}^{h*})y_{hi}^{m}$ and $(1-\beta_{s,hi}^{m*})(1-\beta_{s,hi}^{h*})e_{hi}^{s}$ on the basis of meta-frontier, respectively, in which k = E, B, W; m = U; and s = D, S. On the basis of completing the work above, we then calculated urban total-factor ecological performance index based on the optimal solution. 

Resource consumption control and pollutant emission control are two ways to achieve urban ecological sustainability [13]. Analyzing and comparing these two ecological developments shows how the construction of urban clusters can provide meaningful evidence-based guidelines for policymakers in choosing the best resource conservation measures, allowing them to achieve more effective pollutant emission reductions. In Equation (5), the UTEP is defined as a weighted ratio of the optimal-to-actual resource input and undesirable output in a multi-factor framework. In the absence of any other prior information, the weights assigned to resources and pollutants are determined by equal division. Since the UTEP is a comprehensive evaluation index considering all inputs and outputs (including undesirable outputs), we thus cannot identify its performance in resource consumption and pollutant emission. It is necessary to further decompose UTEP into UTRESP and UTENVP. In Equation (6), following Zhou et al. [33] and Li and Lin [64], we defined the UTRESP as the ratio of the potential target resource intensity to the actual resource intensity—it tries to measure the maximal possible reduction in the resource intensity and represents the degree of resource conservation. Similarly, in Equation (7), the UTENVP is the ratio of the potential target pollutant emission intensity to the actual pollutant emission intensity—it tries to measure the maximal possible reduction in the pollutant intensity and represents the degree of environmental friendliness. The difference between UTRESP and UTENVP is that each of them represents one of the two dimensions in UTEP. The UTEP yields a value between 0 and 1.

(5) UTEP(r,y,e)=UTRESP+UTENVP=UTEP(r,y)+UTEP(e,y)

(6) $$\begin{array}{ll} UTEP(r,y) & =\frac{1}{2}\left[\frac{1}{3}\sum_{k=1}^{3}\frac{(1-\beta_{k,hi}^{m*})(1-\beta_{k,hi}^{h*})r_{hi}^{k}/(1+\beta_{U,hi}^{m*})(1+\beta_{U,hi}^{h*})y_{hi}^{U}}{r_{hi}^{k}/y_{hi}^{U}}\right]\\ & =\frac{1}{2}\left[\frac{1}{3}\sum_{k=1}^{3}\frac{\left(1-\beta_{k,hi}^{m*})(1-\beta_{k,hi}^{h*}\right)}{\left(1+\beta_{U,hi}^{m*})(1+\beta_{U,hi}^{h*}\right)}\right] \end{array}$$

(7) $$\begin{array}{ll} UTEP(e,y) & =\frac{1}{2}\left[\frac{1}{2}\sum_{s=1}^{2}\frac{(1-\beta_{s,hi}^{m*})(1-\beta_{s,hi}^{h*})e_{hi}^{s}/(1+\beta_{U,hi}^{m*})(1+\beta_{U,hi}^{h*})y_{hi}^{U}}{e_{hi}^{s}/y_{hi}^{U}}\right]\\ & =\frac{1}{2}\left[\frac{1}{2}\sum_{s=1}^{2}\frac{\left(1-\beta_{s,hi}^{m*})(1-\beta_{s,hi}^{h*}\right)}{\left(1+\beta_{U,hi}^{m*})(1+\beta_{U,hi}^{h*}\right)}\right] \end{array}$$

### 3.2. The Empirical Strategy

#### 3.2.1. The Impact of Urban Clusters on Resource Conservation and Environmental Friendliness

This article focuses on the ways in which urban clusters affect total-factor ecological performance. To minimize the effects of heteroscedasticity on model estimation, all variables are taken as logarithms, and the dynamic panel regression model is set as follows:

(8) $$\ln S_{it}=\alpha \ln IC_{it}+\rho \ln S_{it-1}+\lambda Z_{it}+\theta_{i}+\nu_{t}+\varepsilon_{it}$$

where $IC_{it}$, representing the degree of urban clustering, is an explanatory variable of interest in this article; $S_{it}$ can the UTENVP, UTRESP, or UTEP; and the subscripts i and t represent cities and years, respectively. In addition, the first-order lag term $S_{it-1}$ of the explained variable is included in the regression model, which is used to characterize the impact of the previous UTEP on that of the current period. $Z_{it}$ are the other control variables, $\theta_{i}$ is city effect, $\nu_{t}$ is time effect, and $\varepsilon_{it}$ captures statistical noise. 

In view of the dual impact of urban clusters on total factor ecological performance, we added the square term to the model to test the possible non-linear relationship between them:

(9) $$\ln S_{it}=\alpha \ln IC_{it}+\beta {\left(\ln IC_{it}\right)}^{2}+\rho \ln S_{it-1}+\lambda Z_{it}+\theta_{i}+\nu_{t}+\varepsilon_{it}$$

#### 3.2.2. Assessing the Industrial Structure Restructuring Effect of Urban Clusters

Urban clusters are conducive to the adjustment of industrial structure and thus affect the development of local economy and ecological environment. To test the effects of urban clusters on industrial structure adjustment, we employed the empirical model as follows:

(10) $$\ln IND_{it}=\omega \ln IC_{it}+\rho \ln IND_{it-1}+\lambda Z_{it}+\theta_{i}+\nu_{t}+\varepsilon_{it}$$

(11) $$\ln S_{it}=\delta_{1}\ln IC_{it}+\rho \ln S_{it-1}+\lambda Z_{it}+\theta_{i}+\nu_{t}+\varepsilon_{it}$$

(12) $$\ln S_{it}=\delta_{2}\ln IC_{it}+\gamma \ln IND_{it}+\rho \ln S_{it-1}+\lambda Z_{it}+\theta_{i}+\nu_{t}+\varepsilon_{it}$$

In Equation (10), *IND* represents the industrial structure supererogation (INDSUP) and industrial structure rationalization (INDRAT), respectively. Equation (10) explores the relationship between urban clusters and industrial structure (mediator variable). If ω is significantly positive, it means the urban clusters have a positive effect on industrial structure adjustment; otherwise, the opposite is the case. Estimating Equations (11) and (12), in the case where $\delta_{1}$, ω and γ are all statistically significant, indicates that the mediation effect exists. On this basis, if $\delta_{2}$ is less than $\delta_{1}$ and both are statistically significant, it appears as a partial mediation effect, that is, urban clusters can directly affect ecological performance, and can also influence ecological performance by promoting the adjustment of industrial structure. 

#### 3.2.3. Assessing the Technological Innovation and Diffusion Effects of Urban Clusters

The spatial agglomeration and networks of urban clusters provide conditions for technological innovation and diffusion, which is an effective way for cities to realize resource conservation and environmental friendliness. We followed Edwards and Lambert [65] and employed the empirical model as follows:

(13) $$\ln TECH_{it}=\beta \ln IC_{it}+\rho \ln TECH_{it-1}+\lambda Z_{it}+\theta_{i}+\nu_{t}+\varepsilon_{it}$$

(14) $$\ln S_{it}=\alpha_{1}\ln IC_{it}+\alpha_{2}\ln TECH_{it}+\alpha_{3}\ln IC_{it}\times \ln TECH_{it}+\rho \ln S_{it-1}+\lambda Z_{it}+\theta_{i}+\nu_{t}+\varepsilon_{it}$$

where β and $\alpha_{2}$ together represent the impact of urban clusters on UTEP through technological innovation. The coefficient $\alpha_{3}$ indicates the effect of urban clusters on technology diffusion. When $\alpha_{3}$ is positive, the higher the degree of clustering, the more conducive it is to technology diffusion; otherwise, the opposite is the case. Additionally, technological innovation and diffusion capabilities may be different in cities with different cluster degrees. Cities with a high cluster degree have a denser population, and are more closely connected with surrounding cities; thus, the agglomeration and network effects of high-cluster cities become stronger. On the contrary, cities with a low cluster degree have a small population scale, which is not conducive to agglomeration and network effect. Therefore, we expect differences in the size and significance of the coefficients $\alpha_{3}$ and β for different cluster degrees.

## 4. Data description and Variable Definitions

### 4.1. Data and Input–Output Variables Used for UTEP and Its Decomposition Index

On the basis of the concept of total factor ecological performance, we selected input–output indicators from three aspects: economy, resources, and the environment. This is taking into consideration that the statistical caliber of some indicators, such as industrial electricity consumption, were changed in 2017, and that several key variables are unavailable before 2004. Therefore, we employed panel data of 278 prefecture-level cities covering the period between 2004 and 2016. All data were obtained from EPS-China (www.epsnet.com.cn) and the China City Statistical Yearbook (2005–2017). The input and output variables and data are detailed in Table 1.

### 4.2. Data and Variable Used for Econometric Model

#### 4.2.1. Measuring the Urban Clustering Degree

There are two main measures to investigate the degree of urban clusters in existing studies. The first is to take the total population of all cities within a given spatial range as a proxy [68]. The second is to increase the location characteristics of cities while considering the urban population [16,69]. In other words, the ratio of population size to urban “remoteness” (*IR*) is taken as a comprehensive index of urban clustering degree, with this index taking full account of the urban population size and spatial factors. In this study, we employed the second measure method, and further added the economic characteristics of cities to reflect the degree of urban clusters more comprehensively. The calculation formula is shown as Equation (15):

(15) $$IC_{it}=\frac{IS}{IR}=\frac{\sum_{j=1}^{n}\sqrt{P_{jt}\times E_{jt}}}{IR_{ik}}$$

where *IS* can be obtained by the sum of the geometric mean values of population size (*P*) and economic size (*E*) of each city within an urban cluster, and the *IR* of a city is measured by its geographical distance from the nearest central city. The definition of the central city and the cluster spatial range are the key factors affecting the degree of urban clusters. We defined the central city as not only meeting the population size of more than 1.5 million, but also ranking as one of the top two economic scales in its province [16,26]. The cluster spatial range was set as an area with a radius of 150 km from the measured city. Additionally, the distance between cities was obtained from map data from the National Geomatics Center of China. The data of population size and economic size was collected from EPS-China data.

#### 4.2.2. Measuring the Industrial Structure Index

Industrial structure supererogation and industrial structure rationalization are two core dimensions to describe the characteristics of industrial development. We calculated the ratio of tertiary industry to secondary industry to reflect INDSUP. The larger the value, the higher the level of INDSUP. The INDRAT is represented by the inverse of the Thiel index [70,71] as follows:

(16) $$INDRAT=1/TL=1/\sum_{i=1}^{n}\left(Y_{i}/Y\right)\ln(\frac{Y_{i}/L_{i}}{Y/L})$$

where *i* is the specific industry, *n* is the number of industries, *Y* is the output, and *L* is the number of labors. The INDRAT is a reflection of the coordination abilities between industries, as well as a measurement of the degree of coordination between factor input and output structure. When *TL* is close to 0, the industrial structure tends to be reasonable. Correspondingly, the larger the *TL*, the more unreasonable the industrial structure.

#### 4.2.3. Other Explanatory Variables

There are many factors affecting the ecological environment, and thus we selected a group of control variables commonly used in the existing literature: (1) Technological level (TECH)—energy intensity is one way to reflect technology, and the electricity intensity can be a proxy variable for technical level [27]; the more GDP per unit of electricity consumption, the higher the level of technology. (2) Industrial structure (IND)—the secondary industry has included a large number of industries with high energy consumption and pollution, while the tertiary industry is mainly composed of clean industries. Therefore, changes in the proportion structure of the industry will affect the quality of the ecological environment. We employed the proportion of the tertiary industry as the proxy variable [26]. (3) Foreign direct investment (FDI)—foreign investment is an important force for China’s economic development. On one path, foreign-invested enterprises with higher energy-saving and emission-reduction technologies can help to carry out more “green” production activities [72]; on another, the pollution paradise hypothesis believes that foreign investment has a negative impact on the environment in developing countries [73]. We used the ratio of the gross output value of foreign-invested industrial enterprises (designed size enterprises) to the gross industrial output value to reflect the level of foreign investment [74]. (4) Environmental regulation (ER)—the impact of ER on urban energy consumption and pollution emissions has been widely demonstrated [75], and this impact causes market players to regulate their actions to reduce the risks of business practice. Due to the availability of data, we used the removal rate of industrial SO_2_ as a proxy variable. (5) Economic development (PERGDP)—economic development is considered to be a factor affecting the quality of the ecological environment [76]. We used real per capita GDP as a proxy variable. Summary statistics of all the above variables are shown in Table 2.

## 5. Results and Discussion

### 5.1. Comparing the UTEP between Low-Cluster Cities and High-Cluster Cities

**Figure 2:**
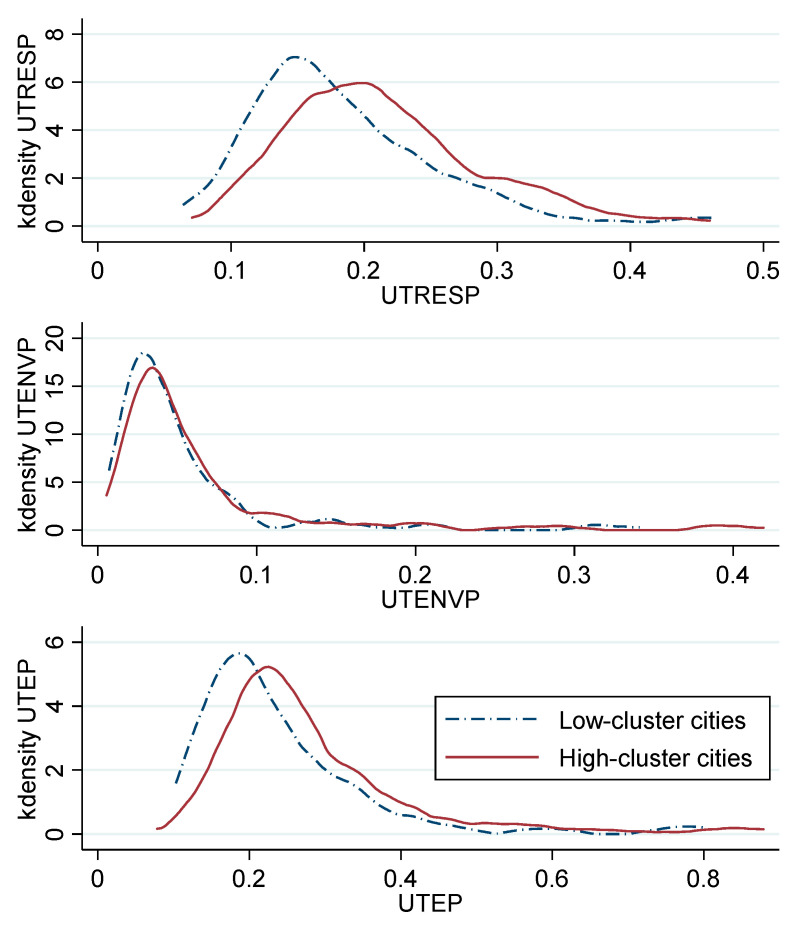
Kernel density curves of UTEP, UTRESP, and UTENVP.

This study divided all cities into high-cluster groups and low-cluster groups on the basis of the degree of urban cluster, so as to visually compare the differences of UTEP between the two groups. Cities with an average degree of urban cluster above the 50% quantile were defined as “high-cluster cities”, and the rest were defined as “low-cluster cities”. Figure 2 plots the kernel density curves of UTEP, UTRESP, and UTENVP of the two groups (see the values in Appendix A). It shows that the kernel density curves of the two groups of cities are quite different. The three kernel density curves of cities with high degree of urban cluster are generally to the right of cities with low degree, which demonstrates that the UTEP of high clustering cities is higher than that of low clustering cities and indicates that urban cluster may be an important factor affecting UTEP. This result also shows that our hypothesis is reasonable and meaningful, but the logic behind the formation of the gap requires further analysis.

### 5.2. Basic Regression Results

We estimated Equation (8) to test the effects of urban clusters on urban resource conservation and environmental friendliness. Considering that the measurement of the explained variables included input–output factors such as capital, labor, and GDP, these factors may be endogenous due to their correlation with explanatory variables. Therefore, this paper used the method of Chen and Golley [77] to estimate the results by using the system-GMM proposed by Arellano and Bover [78]. The system-GMM, to some extent, can address the potential endogeneity bias and eliminate the test error caused by endogenous problems. To prove the robustness of the estimation results, we additionally provide estimation results of two-way fixed effect. The estimated results are reported in Table 3.

In Table 3, columns (1) and (3) report the estimation results of the urban clusters on urban total-factor resource performance (UTRESP) and urban total-factor resource performance (UTENVP) using sys-GMM, respectively. In column (1), the lnIC coefficient and that of its quadratic term are significantly positive at the level of 5% and above, respectively, revealing that the effects of urban clusters on UTRESP have an inverted U-shaped structure. In other words, as the clustering level increases, the UTRESP increases first and then decreases. This may indicate that with the increase of the degree of urban clusters, urban congestion and vicious competition have emerged, which has offset the scale effect and network effect of urban cluster. The lnIC coefficient and that of its quadratic term in column (3) are both positive at a significance level of 5%. This shows that the effects of the urban cluster on the UTENVP present a U-shaped structure, that is, as the level of urban clusters increases, the UTENVP decreases first and then increases. This shows that as the clustering level exceeds a certain threshold, the speed of economic expansion exceeds the speed of environmental degradation.

To demonstrate the robustness of the estimated results, we provide two-way fixed effect estimated results as a comparison. The first is the coefficients comparison of ${\left(\ln IC\right)}^{2}$, wherein columns (2), (4), and (6) of Table 3 provide the estimated results of the two-way fixed effect as a robust analysis, and the signs of ${\left(\ln IC\right)}^{2}$ are respectively the same as the results of columns (1), (3), and (5). The second is the coefficients comparison of lnIC, although the signs in columns (1) and (2) are opposite, the signs in columns (3)–(6) are the same, and the judgment of the inverted U-shaped relationship between urban cluster and UTRESP is not affected. The last is the coefficients comparison of other control variables; we note that the coefficients of other variables are basically the same except for some insignificant estimates. At the same time, another strategy was provided to compare changes in estimated results by reducing a control variable, including subsequent Section 5.3 and Section 5.4, which involve robust analysis, and we obtained basically consistent estimated results. Due to space limitations, the related estimated results are shown in Appendix B, Appendix C and Appendix D. Both of the methods above can prove the robustness of the estimated results.

Comparing the estimation results of columns (1) and (3) of Table 3, the effect trend of urban clusters on UTRESP and UTENVP is opposite (inverted U-shaped and U-shaped), which shows that with the improvement of urban clusters, the corresponding cities gradually change from resource-saving cities to environmental-friendly cities, and the latter may become the main way to promote the improvement of the overall UTEP. Columns (5) and (6) provide the estimation results of overall effects of urban clusters on UTEP. The lnIC coefficient is positive at a significance level of 1%, and that of its quadratic term is not statistically significant, indicating that the urban cluster will eventually promote the continuous improvement of UTEP (sum of UTRESP and UTENVP). This is consistent with the conclusion of Huang et al. [26]. The possible reason is that when the urban clustering degree exceeds a certain threshold, the inhibitory effects on the UTRESP is offset by the promotion effects on the UTENVP, and finally promotes the improvement of the UTEP.

The reasons for the results mentioned above are as follows. On the one hand, China has been undergoing a rapid and intensive processes of urbanization. In the process of urban agglomeration construction, cities have been offering cheap production resources, such as land resources, for the purpose of competing for capital entry, causing distortions in resource allocation. In addition, the extensive development model relying on resource input has formed a large amount of excess production capacity, leading to the existence of economically inefficient output. On the other hand, it is related to the scale effect and network externality of urban cluster. This may help to improve energy efficiency and the quality of the environment. Meanwhile, it is also related to the collaborative system of environmental governance of urban cluster. For example, the “12th Five-Year Plan for the Prevention and Control of Air Pollution in Key Regions” requires the establishment of a regional air pollution joint prevention and control mechanism in China’s three major urban agglomerations, so as to realize the adjustment of industrial layout on the basis of considering environmental carrying capacity and resource endowment. In addition, the enhancement of citizens’ awareness of environmental protection and the participation of social forces eventually achieve the continuous improvement of environmental quality.

### 5.3. Industrial Structure Restructuring Effect

Industrial structure restructuring is another important line to understand the effect of urban clusters. As shown in Table 4, columns (1) and (6) test the effects of urban clusters on the supererogation and rationalization of industrial structure (INDSUP and INDRAT). The coefficients of lnIC are positive at a significance level of 5% and 1% (on the basis of Equation (10)), respectively, indicating that urban clusters have promoted the supererogation and rationalization of the industrial structure, similar to the conclusion of Q. Yuan. [16]. We now explore how urban clusters influence the UTRESP and UTENVP through industrial structure adjustment. First, columns (2)–(5) report the impact of INDSUP as a mediator variable on UTRESP and UTENVP. The coefficient of lnIC in columns (2) and (3) are statistically significant at the level of 1% (on the basis of Equations (11) and (12)), but the lnINDRAT coefficient in column (3) does not pass the significance test. It reveals that supererogation of industrial structure, the mediator variable, only has a positive on UTENVP, but not on UTRESP. The lnIC coefficient in columns (4) and (5) are both positive at a significance level of 5%, and the coefficient that in column (5) positively pass the significance level of 10% (on the basis of Equation (12)). It indicates that the supererogation of industrial structure has a positive effect on UTENVP. Second, similarly, columns (7)–(10) report the impact of INDRAT as a mediator variable on UTRESP and UTENVP. The estimated coefficient of lnINDRAT in column (10) is positive at the significance level of 5% (on the basis of Equation (12)), indicating that urban clusters also promote the improvement of UTENVP rather than UTRESP through the rationalization of industrial structure. Third, we observed the estimated coefficients of lnIC in columns (4) and (5)—it becomes smaller after lnINDSUP is added into the corresponding equation. This is because that the supererogation of industrial structure as a transmission channel disperses a part of the direct effect of urban clusters. The estimated results of columns (9) and (10) illustrate a similar phenomenon.

On the basis of the results estimated above, we can conclude that urban clusters on adjustment of the industrial structure only produce an environmental friendliness effect and do not produce a resource conservation effect. It may be that clusters help central cities attract “environmentally friendly” industries, increasing the share of tertiary industries, such as producer services and high-tech industries, and thus reducing the proportion of polluting industries. At the same time, through the close relationship with the central city, the peripheral cities undertake a part of the high-quality enterprises that move out due to the congestion effect in central cities, improving the level of industrial rationalization, so as to improve the total-factor ecological performance.

### 5.4. Technology Innovation and Diffusion Effect

The technology innovation and diffusion of urban clusters are effective ways to achieve the resource conservation and environmental friendliness effects. We focused on the coefficients of the interaction term lnIC×lnTECH (based on Equation (13)) and the coefficients of lnIC (based on Equation (14)). Since both of the two coefficients pass the significance test, it means that the urban cluster has affected the urban ecological environment through technology innovation and diffusion. To examine the differences in technology innovation and diffusion at different clustering levels, we additionally report the grouped regression results of Equations (13) and (14). In Table 5, the coefficients of lnIC in columns (1) and (3) are all positive at the significance level of 1%, while the coefficients of of lnIC in column (2) is not statistically significant, indicating that urban clustering stimulates technological innovation only when the clustering level exceeds a certain threshold. Then, we discuss how urban cluster can improve UTRESP and UTENVP through technology innovation and diffusion.

Columns (4)–(6) report the impact of technology innovation and diffusion on UTRESP. Whether they are the estimated results of the full sample or those of the grouped sample, the coefficients of lnTECH are significantly positive, and the coefficients of lnIC×lnTECH are not statistically significant. These results indicate that technology innovation effect is an important way to improve UTRESP, but technology diffusion effects have not appeared. Columns (7)–(9) report the impact of technology innovation and diffusion on UTENVP. The signs of lnTECH and lnIC×lnTECH in column (7) are both positive at the significance level of 1%, and the partial differential equation is ∂(lnUTENVP)/∂(lnTECH)=(0.535+0.077lnIC). In this equation, only if lnIC≥−6.94 (IC≥0.001) may technology diffusion affect the UTENVP positively, and the higher the level of urban clusters, the stronger its promotion effect on environmental friendliness. Columns (8) and (9) report the grouped estimation results on the basis of the clustering level, wherein we noticed that the coefficient of the interaction term is not statistically significant when the clustering level is below 50%; correspondingly, the coefficient of the interaction term is significantly positive when the clustering level exceeds 50%. Meanwhile, the coefficient of the latter is greater than the estimated coefficient of the full sample, and the coefficient of lnTECH is also significantly positive. It further reveals that cities with higher clustering level are more likely to have the technology innovation and diffusion effect, which is a concrete manifestation of the externality of urban cluster networks. In summary, within urban clusters, the technological innovation effect achieves the double promotion of resource conservation and environmental friendliness, while the technological diffusion effect only achieves environmental friendliness without providing promotion to resource conservation.

## 6. Heterogeneity Analysis

### 6.1. The Estimated Results of Region Grouping

In Table 6, the coefficients of lnIC in columns (1) and (2) are significantly positive, while the those in columns (5) and (6) are not statistically significant, indicating that the urban clusters in the eastern region have exerted a positive effect on UTRESP and UTENVP, thus promoting the improvement of UTEP simultaneously in these two dimensions; correspondingly, western urban clusters have not played an active role of urban clusters in resource utilization and environmental quality improvement. The coefficient of lnIC in column (3) is only positive at the significance level of 10%, while that in column (4) is not statistically significant, showing that the urban clusters in the central region only have a weak UTRESP effect, but no UTENVP effect. Therefore, in the central region, the promotion of UTEP by urban cluster is very limited.

Urban clusters in the eastern region are relatively mature, and the city network is more closely connected, which facilitates cities learning and catching up with each other, thus improving the level of technology and effectively bringing into play the scale effect and network effect. As shown in Appendix A, UTRESP and UTENVP rank among the top two cities in the eastern coastal regions and are eventually reflected in the UTEP ranking. The urban clusters in the central and western regions, especially in the western region, are in the process of development, and they are in a more competitive relationship, resulting in serious local protectionism. The flow of factors between cities is not free enough, and the benign interaction of talents is less, and thus there is a lack of complementary advantages and technology exchange. Urban clusters do not play a radiation capacity, and the industrial structure is backward, resulting in the slow improvement of resource efficiency and environmental quality. This is why the UTEPs of cities in the central and western regions rank low in Appendix A. Therefore, urban clusters in the central and western regions have great potential space to achieve resource conservation and environmental friendliness.

### 6.2. The Estimated Results of Period Grouping

We divided the entire sample period into two sub-periods of 2004–2009 and 2010–2016. The estimated results are obtained in Table 7. 

The impact of urban clusters on UTRESP and UTENVP differed significantly in two periods. In the period of 2004–2009, the coefficients of lnIC in columns (1) and (3) both failed the significance test. In the period of 2010–2016, the coefficients of lnIC in columns (2) and (4) were significantly positive at the level of 5% and 1%, respectively, and the sign was positive, indicating that urban clusters can significantly improve UTRESP and UTENVP. This may be attributed to the great improvement in the level and quality of urban clusters in the latter half of the stage, which was more effective in improving the level of resource utilization and pollution reduction, especially the latter. Therefore, along with the continuous construction of urban cluster, its role in improving the total factor ecological performance is gradually manifested, especially in the environmentally friendly dimension.

## 7. Conclusions and Implications

### 7.1. Conclusions

Urban cluster is an important manifestation of the spatial agglomeration of economic activities, and an important way to accelerate the urbanization process in China. On the basis of the theory of agglomeration economy and spatial externality, this study investigated the potential impact of urban clusters on the regional ecological environment. We constructed an urban cluster index by utilizing the panel data of 278 cities in China during 2004–2016. To study the ways in which urban clusters influence the UTEP, we decomposed it into the UTRESP and the UTENVP under a unified measurement framework based on an extended meta-frontier model, and took them as measures of resource conservation and environmental friendliness. Using the empirical analysis method of the dynamic panel regression model, we revealed the role of urban clusters in affecting resource conservation and environmental friendliness. As a large-scale spatial agglomeration, urban clusters exerted both a spatial agglomeration effect and a network effect. With the increase of the urban clustering degree, the corresponding cities were more likely to change from resource-saving cities to environmentally friendly cities. Environmental friendliness became the main way for urban clusters to promote the improvement of UTEP. The estimated results of region grouping showed that due to the relatively mature development of urban clusters in the eastern region, cities within the urban clusters were closely linked, forming a benign interaction and improving the UTEP. However, urban clusters in the western–central regions of China were in the process of growth, and the relationship between cities was more competitive with serious local protection. Moreover, the phenomenon of “congestion effect” in central cities and “brain drain” in peripheral cities existed simultaneously, which was not conducive to forming a cluster effect.

We can summarize the results into three aspects from the perspective of the effect mechanisms. First, urban clusters exerted clustering innovation effects to accelerate knowledge spillovers and technology diffusion. Technological innovation and diffusion significantly improved the UTEP, and the higher the clustering degree was, the stronger the improvement of UTENVP, thus making environment friendliness an important “fruit” of urban clusters. Second, urban clusters facilitated the division of labor and industrial layout in a larger area, and expanded cooperation and exchanges between central cities and peripheral cities; moreover, they promoted the optimization of industrial structure, alleviating the urban crowding effect, and ultimately improving the TUEP. Third, urban clusters expanded the scale of production and consumption, forming the effects of scale economy, which directly improved the utilization efficiency of resources and provided conditions for the improvement of UTEP.

### 7.2. Implications

On the basis of the above research conclusions, we have six points of implications: (1) Improving the quality of urban cluster construction. We need to transform the traditional single-point development model to the multi-point networked development model on the issue of urbanization development, as well as strengthen the infrastructure construction, such as transportation and telecommunication network within urban clusters, in order to facilitate the connection between central cities and peripheral cities. This will give full play to the network effect, agglomeration effect, and scale effect of the urban clusters. (2) Governing urban clusters in terms of resource conservation and environmental friendliness. One way is to prevent vicious competition within urban clusters and improve the efficiency of urban construction land and energy utilization through perfect market-oriented means and supplement it by policy guidance, so as to reduce resource misallocation. The other is to expand the coverage of the joint prevention and control mechanism of pollutant emission in urban clusters and improve the effectiveness of environmental regulation policies. (3) Scientific planning and policy guidance for the construction of urban clusters. To illustrate, we can attempt the construction model of new urban clusters in the Guangdong–Hong Kong–Macao Greater Bay Area, give full play to the radiation effect of the relatively mature Yangtze River Delta urban agglomeration and the Bohai Rim urban agglomeration, and attract resources to the developing urban clusters in the Chengdu–Chongqing urban agglomeration and the Central Henan urban agglomeration, so as to take advantage of urban agglomerations in terms of resource utilization and pollution control. (4) Making use of the technological innovation and technology diffusion effect of urban clusters. In one path, we can regard the central city as the innovation center to improve the technology spillover on the surrounding cities, in another, the barriers of communication and cooperation between cities should be removed, and the driving force of technological progress and sharing for urban sustainability should be brought into play. (5) Good industrial division and cooperation should be achieved within urban clusters. Big cities (central cities) can attract clean industries, knowledge-intensive industries, and service business, as well as optimize their industrial structures. Small and medium-sized cities (peripheral cities) can develop industries with their own endowment advantages, thus rationalizing the industrial structure and promoting the improvement of resource and environmental efficiency. (6) More attention needs to be paid to the non-clusters. As mentioned in this article, there is much space for improvement of UTRESP and UTENVP in cities with low clustering level. Therefore, it will be particularly important to explore how to correctly coordinate the development of the cities within and outside the clusters and avoid any gaps between them.

This article expands the measurement and decomposition of total factor ecological performance, and also makes a new study on the transmission channels between the urban clusters and total-factor ecological performance (UTEP). However, there are still some deficiencies in this article and areas for further study. First, the research perspective can be further expanded, for example, when the UTREP and UTENVP are decomposed with respect to each input-output variable, the research on UTEP can provide more information. In addition, the Malmquist–Luenberger index can also be used to analyze the impact of urban clusters on UTEP growth from the perspective of technological efficiency and technological progress. Second, the transmission channels between urban cluster and UTEP need to be further expanded, such as market integration and population migration. These deficiencies and research suggestions mentioned above can help refine and deepen the research objectives and support policymakers to formulate more targeted policy strategies.

## Figures and Tables

**Table 1:** Input-output variables and data description.

Category	Variables	Data and Description
total factor inputs	Labor(*L*)	We use total employment at year-end as the labor input.
	Capital(*K*)	The “perpetual inventory method” is adopted to calculate the capital stock. We converted all the fixed asset investment data to 2004 prices using price indices for investment in fixed assets by region.
	Land resources(*B*)	We use the urban built-up area as the land resources input [27].
	water resources (*W*)	We subtract the domestic water consumption from the total water supply to get the water input.
	Energy (*E*)	As the energy consumption data is not available at the city level, we use industrial electricity consumption as a proxy indicator for its high correlation with energy consumption [64].
Desirable output	GDP(U)	To account for the price effect on economic data, we converted it to 2004 prices.
Undesirable output	Waste water(*D*)	We use industrial wastewater emission as waste water.
	Waste gas (*S*)	Considering the issue of statistical caliber, we use industrial sulfur dioxide emission as waste gas.

Notes: The calculation formula of $K_{it}$ is $I_{it}+(1-\delta_{t})K_{it-1}$, in which $I_{it}$ is the fixed asset investment of the city *i* in year *t*. We calculated the capital stock ($K_{it-1}$) in the initial year by using 10 times the fixed asset investment in 1996 [66]. The depreciation rate ($\delta_{t}$) can be obtained by referring to Xiang [67]. The statistical caliber of industrial smoke (dust) as waste gas during the sample period is inconsistent, so this variable is not used as a component of undesirable output. All data is obtained within the municipal district, except for waste water and waste gas, which are only collected from whole city by the Bureau of Statistics.

**Table 2:** Summary statistics.

Variables	Obs.	Mean	SD	Min	Max
Urban total-factor ecological performance (UTEP)	3614	0.260	0.154	0.053	0.999
Urban total-factor resource performance (UTRESP)	3614	0.201	0.090	0.034	0.500
Urban total-factor environmental performance (UTEVNP)	3614	0.059	0.080	0.001	0.500
Urban clusters (IC)	3614	0.378	1.257	0.000	12.672
Technological level (TECH)	3614	0.109	0.100	0.004	1.649
Industrial structure (IND)	3614	0.424	0.110	0.086	0.802
Foreign direct investment (FDI)	3614	0.173	0.182	0.000	0.919
Environmental regulation (ER)	3614	0.454	0.272	0.000	0.994
Economic development (PERGDP)	3614	0.108	0.049	0.021	0.521
Industrial structure supererogation (INDSUP)	3614	0.952	0.541	0.094	4.819
Industrial structure rationalization (INDRAT)	3614	0.451	2.205	0.005	67.284

**Table 3:** Basic estimated results.

	UTRESP		UTENVP		UTEP	
Variables	(1)	(2)	(3)	(4)	(5)	(6)
	System-GMM	Two-Way	System-GMM	Two-Way	System-GMM	Two-Way
ln*IC*	−0.033 **	0.405 ***	0.163 ***	1.049 ***	0.339 ***	0.533 ***
	(−2.02)	(8.16)	(3.12)	(6.13)	(3.16)	(8.39)
(ln*IC*)^2^	−0.006 ***	−0.011 ***	0.019 **	0.058 ***	0.003	0.003
	(−2.80)	(−4.12)	(2.46)	(6.11)	(0.46)	(0.81)
ln*TECH*	0.223 ***	0.301 ***	0.173 ***	0.335 ***	0.323 ***	0.318 ***
	(12.28)	(31.89)	(4.22)	(10.27)	(15.75)	(26.30)
ln*IND*	−0.039	0.041	−0.022	0.055	−0.175 **	0.064 *
	(−1.04)	(1.48)	(−0.18)	(0.59)	(−2.59)	(1.83)
ln*FDI*	0.004	0.026 ***	−0.038 *	0.029	0.017	0.027 ***
	(0.59)	(4.40)	(−1.79)	(1.39)	(1.38)	(3.46)
ln*ER*	−0.004	0.001	−0.001	0.018	−0.000	0.004
	(−0.82)	(0.18)	(−0.07)	(1.27)	(−0.03)	(0.82)
ln*PERGDP*	0.299 ***	0.261 ***	1.013 ***	1.210 ***	0.320 ***	0.417 ***
	(11.10)	(16.15)	(12.08)	(21.71)	(6.54)	(20.15)
L.ln*UTRESP*	0.376 ***					
	(8.22)					
L.ln*UTENVP*			0.343 ***			
			(7.39)			
L.ln*UTEP*					0.254 ***	
					(5.29)	
Time effect	Yes	Yes	Yes	Yes	Yes	Yes
City effect	Yes	Yes	Yes	Yes	Yes	Yes
*R^2^*		0.490		0.328		0.457
*F*	131.066	167.443	81.637	85.171	50.658	147.056
Hanse	0.257		0.326		0.312	
AR(2)	0.832		0.640		0.496	
Observations	3336	3614	3336	3614	3336	3614

Notes: The *t*-statistics for the coefficients in parentheses ***, **, and * are for 1%, 5%, and 10% significance levels, respectively.

**Table 4:** Industrial structure restructuring effect.

	INDSUP	UTRESP		UTENVP		INDRAT	UTRESP		UTENVP	
Variables	(1)	(2)	(3)	(4)	(5)	(6)	(7)	(8)	(9)	(10)
ln*IC*	0.216 **	0.022 ***	0.016 *	0.056 **	0.053 **	0.440 ***	0.022 ***	0.023 ***	0.048 *	0.045 *
	(2.45)	(2.65)	(1.80)	(2.26)	(2.15)	(2.78)	(2.65)	(2.93)	(1.83)	(1.87)
ln*INDSUP*			−0.028		0.146 *					
			(−0.98)		(1.89)					
ln*INDRAT*								−0.013		0.064 **
								(−1.34)		(2.46)
L.ln*UTRESP*		0.484 ***	0.425 ***				0.484 ***	0.485 ***		
		(12.74)	(13.21)				(12.74)	(13.07)		
L.ln*UTENVP*				0.441 ***	0.433 ***				0.420 ***	0.416 ***
				(11.58)	(11.35)				(12.12)	(11.68)
Control variable	Yes	Yes	Yes	Yes	Yes	Yes	Yes	Yes	Yes	Yes
Time effect	Yes	Yes	Yes	Yes	Yes	Yes	Yes	Yes	Yes	Yes
City effect	Yes	Yes	Yes	Yes	Yes	Yes	Yes	Yes	Yes	Yes
*F*	63.808	86.328	67.409	87.774	85.838	13.701	86.328	88.272	44.779	43.660
Hanse	0.180	0.145	0.102	0.315	0.303	0.326	0.145	0.260	0.396	0.363
AR(2)	0.354	0.806	0.964	0.411	0.424	0.887	0.806	0.741	0.448	0.484

Notes: The sample size (no. of observations) is 3336; the *t*-statistics for the coefficients in parentheses ***, **, and * are for 1%, 5%, and 10% significance levels, respectively.

**Table 5:** Technology innovation and diffusion effect.

Variables	TECH			UTRESP			UTENVP		
	(1)	(2)	(3)	(4)	(5)	(6)	(7)	(8)	(9)
	Full	Ic ≤ 50%	Ic > 50%	Full	Ic ≤ 50%	Ic > 50%	Full	Ic ≤ 50%	Ic > 50%
ln*IC*	0.049 **	0.006	0.109 ***	0.224 ***	0.187 *	0.305 ***	−0.189 **	2.366 *	0.342
	(2.31)	(0.12)	(3.11)	(3.03)	(1.78)	(2.90)	(−2.05)	(1.79)	(0.67)
ln*TECH*				0.343 ***	0.459 ***	0.360 ***	0.535 ***	−0.408	1.062 ***
				(7.10)	(4.14)	(7.53)	(3.72)	(−0.67)	(4.93)
Ln*IC* × ln*TECH*				0.009	0.028	0.016	0.077 ***	−0.096	0.178 ***
				(0.82)	(1.51)	(1.14)	(2.60)	(−1.28)	(2.76)
L.ln*TECH*	0.520 ***	0.459 ***	0.597 ***						
	(9.82)	(6.80)	(10.18)						
L.ln*UTRESP*				0.315 ***	0.302 ***	0.310 ***			
				(7.11)	(5.74)	(5.63)			
L.ln*UTENVP*							0.361 ***	0.356 ***	0.320 ***
							(7.76)	(5.27)	(5.52)
Control variables	Yes	Yes	Yes	Yes	Yes	Yes	Yes	Yes	Yes
Time effect	Yes	Yes	Yes	Yes	Yes	Yes	Yes	Yes	Yes
City effect	Yes	Yes	Yes	Yes	Yes	Yes	Yes	Yes	Yes
*F*	28.898	13.032	30.209	57.786	41.484	26.398	71.639	18.467	25.536
Hanse	0.168	0.812	0.756	0.113	0.172	0.409	0.169	0.065	0.230
AR(2)	0.051	0.148	0.152	0.576	0.056	0.941	0.664	0.679	0.872
Observations	3336	1668	1668	3336	1668	1668	3336	1668	1668

Notes: The *t*−statistics for the coefficients in parentheses ***, **, and * are for 1%, 5%, and 10% significance levels, respectively.

**Table 6:** Estimated results of eastern, central, and western cities.

	East		Central		West	
Variables	(1)	(2)	(3)	(4)	(5)	(6)
	UTRESP	UTENVP	UTRESP	UTENVP	UTRESP	UTENVP
Lnic	0.011 **	0.052 **	0.009 *	0.025	−0.005	0.049
	(1.96)	(2.19)	(1.70)	(1.53)	(−0.71)	(1.40)
L.Lnutresp	0.501 ***		0.444 ***		0.378 ***	
	(7.14)		(5.23)		(3.24)	
L.Lnutenvp		0.355 ***		0.351 ***		0.264 ***
		(5.46)		(5.19)		(3.18)
Control variables	Yes	Yes	Yes	Yes	Yes	Yes
Time effect	Yes	Yes	Yes	Yes	Yes	Yes
City effect	Yes	Yes	Yes	Yes	Yes	Yes
*F*	78.118	37.154	170.784	44.631	56.468	13.337
Hanse	0.251	0.798	0.338	0.307	0.324	0.779
AR(2)	0.914	0.929	0.946	0.486	0.523	0.136
Observations	1380	1380	1308	1308	648	648

Notes: The *t*-statistics for the coefficients in parentheses ***, **, and * are for 1%, 5%, and 10% significance levels, respectively.

**Table 7:** Estimated results of periods of 2004–2009 and 2010–2016.

	UTRESP		UTENVP	
Variables	(1)	(2)	(3)	(4)
	2004–2009	2010–2016	2004–2009	2010–2016
ln*IC*	−0.049	0.056 **	0.025	0.671 ***
	(−1.35)	(2.59)	(1.64)	(4.55)
L.ln*UTRESP*	0.386 ***	0.420 ***		
	(5.07)	(6.89)		
L.ln*UTENVP*			0.371 ***	0.272 ***
			(5.24)	(5.77)
Control variables	Yes	Yes	Yes	Yes
Time effect	Yes	Yes	Yes	Yes
City effect	Yes	Yes	Yes	Yes
*F*	71.942	100.518	47.219	33.398
Hanse	0.202	0.242	0.898	0.181
AR(2)	0.724	0.628	0.387	0.506
Observations	1390	1946	1390	1946

Notes: The *t*-statistics for the coefficients in parentheses ***, and ** are for 1%, and 5% significance levels, respectively.

## Appendix A

According to the measurement method mentioned above, we calculated the UTEP and its decomposition result of 278 prefecture-level cities in China from 2004 to 2016. Taking into account the limitations of typography, we only provide the calculation results of the top 25 and bottom 25 cities (see Table A1).

**Table A1:** The average and decomposition of UTEP of the top 25 and bottom 25 cities from 2004 to 2016.

Top 25				Bottom 25			
Cities	UTEP	UTRESP	UTENVP	Cities	UTEP	UTRESP	UTENVP
Foshan	0.879	0.460	0.419	Yuncheng	0.149	0.137	0.012
Zhongshan	0.844	0.451	0.393	Huainan	0.149	0.130	0.019
Changde	0.803	0.462	0.341	Xuchang	0.148	0.127	0.021
Dongguan	0.799	0.412	0.387	Benxi	0.148	0.106	0.041
Erdos	0.766	0.447	0.319	Hechi	0.147	0.140	0.007
Yuxi	0.765	0.456	0.309	Yichun	0.147	0.097	0.050
Changsha	0.700	0.403	0.297	Hengyang	0.146	0.110	0.037
Shenzheng	0.636	0.377	0.259	Shangrao	0.146	0.124	0.022
Daqing	0.602	0.321	0.281	Baise	0.146	0.124	0.022
Sanya	0.594	0.388	0.206	Liaoyang	0.145	0.100	0.045
Dongying	0.586	0.375	0.211	Zhumadian	0.140	0.122	0.018
Guangzhou	0.553	0.375	0.178	Huangshi	0.138	0.122	0.016
Beijing	0.546	0.339	0.207	Anqing	0.136	0.109	0.027
Hohhot	0.537	0.331	0.206	Jiayuguan	0.135	0.064	0.072
Tianjin	0.501	0.337	0.164	Qitaihe	0.133	0.107	0.026
Putian	0.471	0.360	0.111	Jixi	0.129	0.094	0.035
Qingyang	0.463	0.325	0.138	Zhoukou	0.126	0.113	0.013
Haikou	0.460	0.265	0.196	Hehe	0.124	0.106	0.018
Maoming	0.459	0.324	0.135	Kaifeng	0.122	0.101	0.021
Suozhou	0.438	0.297	0.141	Mudanjiang	0.113	0.083	0.030
Ziyang	0.437	0.333	0.104	Hegang	0.111	0.089	0.022
Qingdiao	0.432	0.350	0.082	Wuzhong	0.106	0.087	0.019
Shangluo	0.430	0.314	0.116	Xingtai	0.103	0.098	0.005
Shengyang	0.420	0.315	0.105	Fuxing	0.103	0.084	0.019
Karamay	0.416	0.261	0.155	Jiaozuo	0.078	0.071	0.008
Mean	0.581	0.363	0.218	Mean	0.131	0.106	0.025
Percent(%)		62.48	37.52	Percent(%)		80.92	19.08

As shown in Table A1, the average UTEP of the top 25 cities is 0.581, while the average UTEP of the bottom 25 cities is 0.131; the former is four times more than that of the latter. Most of the top cities are located in the eastern coastal areas, with a dense network of surrounding cities and a high degree of urban clusters. To illustrate, Foshan and Zhongshan, located in the Guangdong–Hong Kong–Macao Greater Bay Area, are surrounded by many large cities, which are closely connected with each other, whereas the bottom cities are mostly located in the central plains region and northeast region, with sparsely distributed cities and low clustering degree. This indicates that the regional gap of total factor ecological performance is very large, and cities in backward areas such as the northwest have great potential space to achieve resource conservation and environmental friendliness.

From the perspective of the composition of UTEP, the value of UTRESP is higher than that of UTENVP in all types of cities. The latter is the dominant contributor to the evolution of UTEP. In particular, the UTRESP and UTENVP of the top-ranked cities are 62.48% and 37.52%, respectively, with the former slightly higher than the latter. This reveals that the performance of such cities in terms of resource conservation and pollution reduction is relatively close, which makes little difference between the two in terms of their contribution to the urban UTEP. In contrast, although the UTRESP and UTENVP of cities at the bottom of the list both are very low, the proportion of them is very different. The average value (0.106) of UTRESP accounts for 80.92% of the UTEP, while the value of UTENVP (0.025) only accounts for 19.08% of the UTEP—this indicates that there is still a lot of space for pollution reduction in these cities to promote the improvement of UTEP, which may be related to the weak environmental constraints of these cities. Most of these cities are relatively backward with low technical level and weak environmental constraint, which leads to large pollution emissions and affects environmental performance.

## Appendix B

**Table A2:** Robust analysis of basic estimated results.

	UTRESP		UTENVP		UTEP	
Variables	(1)	(2)	(3)	(4)	(5)	(6)
	Sys-GMM	Two-Way	Sys-GMM	Two-Way	Sys-GMM	Two-Way
ln*IC*	−0.033 **	0.416 ***	0.164 ***	1.062 ***	0.345 ***	0.544 ***
	(−1.98)	(8.38)	(3.12)	(6.21)	(3.21)	(8.57)
(ln*IC*)^2^	−0.006 ***	−0.011 ***	0.020 ***	0.059 ***	0.004	0.004
	(−2.89)	(−3.84)	(2.73)	(6.21)	(0.64)	(1.03)
ln*TECH*	0.223 ***	0.301 ***	0.170 ***	0.335 ***	0.324 ***	0.318 ***
	(12.28)	(31.83)	(4.02)	(10.28)	(15.72)	(26.28)
ln*IND*	−0.034	0.052 *	−0.064	0.068	−0.168 **	0.076 **
	(−0.96)	(1.91)	(−0.53)	(0.72)	(−2.50)	(2.17)
ln*ER*	−0.004	0.000	−0.001	0.018	−0.000	0.004
	(−0.81)	(0.09)	(−0.06)	(1.24)	(−0.05)	(0.75)
ln*PERGDP*	0.300 ***	0.259 ***	1.008 ***	1.208 ***	0.321 ***	0.415 ***
	(11.05)	(16.02)	(12.19)	(21.69)	(6.55)	(20.05)
L.ln*UTRESP*	0.378 ***					
	(8.28)					
L.ln*UTENVP*			0.342 ***			
			(7.48)			
L.ln*UTEP*					0.258 ***	
					(5.38)	
Time effect	Yes	Yes	Yes	Yes	Yes	Yes
City effect	Yes	Yes	Yes	Yes	Yes	Yes
*R^2^*		0.487		0.328		0.455
*F*	135.548	174.705	79.388	89.770	52.686	154.049
Hanse	0.262		0.364		0.323	
AR(2)	0.841		0.627		0.506	
Observations	3336	3614	3336	3614	3336	3614

Notes: The *t*-statistics for the coefficients in parentheses ***, **, and * are for 1%, 5%, and 10% significance levels, respectively.

## Appendix C

**Table A3:** Robust analysis of industrial structure restructuring effect.

	Industrial Structure Supererogation					Industrial Structure Rationalization				
	INDSUP	UTRESP		UTENVP		INDRAT	UTRESP		UTENVP	
Variables	(1)	(2)	(3)	(4)	(5)	(6)	(7)	(8)	(9)	(10)
ln*IC*	0.214 ***	0.026 ***	0.017 **	1.347 **	0.035 *	0.413 ***	0.026 ***	0.025 ***	1.732 ***	0.036 **
	(3.84)	(3.00)	(2.04)	(2.14)	(1.66)	(2.72)	(3.00)	(2.91)	(2.81)	(2.20)
ln*INDSUP*			−0.040		0.157 *					
			(−1.32)		(1.97)					
ln*INDRAT*								−0.015		0.052 **
								(−1.34)		(2.24)
L.ln*UTRESP*		0.450 ***	0.414 ***				0.450 ***	0.466 ***		
		(11.95)	(12.47)				(11.95)	(12.93)		
L.ln*UTENVP*				0.337 ***	0.439 ***				0.355 ***	0.551 ***
				(8.47)	(11.86)				(8.66)	(10.93)
Control var.	Yes	Yes	Yes	Yes	Yes	Yes	Yes	Yes	Yes	Yes
Time effect	Yes	Yes	Yes	Yes	Yes	Yes	Yes	Yes	Yes	Yes
City effect	Yes	Yes	Yes	Yes	Yes	Yes	Yes	Yes	Yes	Yes
*F*	95.621	84.813	68.313	37.812	103.593	14.938	84.813	92.975	40.043	68.202
Hanse	0.103	0.228	0.039	0.408	0.187	0.303	0.228	0.358	0.344	0.357
AR(2)	0.363	0.885	0.921	0.686	0.390	0.821	0.885	0.774	0.610	0.257

Notes: The sample size (no. of observations) is 3336, the *t*-statistics for the coefficients in parentheses ***, **, and * are for 1%, 5%, and 10% significance levels, respectively.

## Appendix D

**Table A4:** Robust analysis of technology innovation and diffusion effect.

	TECH			UTRESP			UTENVP		
	(1)	(2)	(3)	(4)	(5)	(6)	(7)	(8)	(9)
Variables	Full	Ic ≤ 50%	Ic > 50%	Full	Ic ≤ 50%	Ic > 50%	Full	Ic ≤ 50%	Ic > 50%
ln*IC*	0.046 **	0.000	0.089 **	0.223 ***	0.180 *	0.292 ***	−0.198 **	2.399 *	0.355
	(2.33)	(0.01)	(2.58)	(3.02)	(1.72)	(2.81)	(−2.10)	(1.81)	(0.70)
ln*TECH*				0.349 ***	0.466 ***	0.363 ***	0.534 ***	−0.439	1.056 ***
				(7.26)	(4.26)	(7.51)	(3.56)	(−0.74)	(4.92)
ln*IC*×ln*TECH*				0.010	0.029	0.017	0.076 **	−0.101	0.177 ***
				(0.92)	(1.59)	(1.16)	(2.47)	(−1.41)	(2.77)
L.ln*TECH*	0.519 ***	0.460 ***	0.601 ***						
	(9.77)	(6.67)	(10.08)						
L.ln*UTRESP*				0.318 ***	0.308 ***	0.315 ***			
				(7.23)	(5.94)	(5.83)			
L.ln*UTENVP*							0.362 ***	0.351 ***	0.321 ***
							(7.84)	(5.17)	(5.53)
Control variables	Yes	Yes	Yes	Yes	Yes	Yes	Yes	Yes	Yes
Time effect	Yes	Yes	Yes	Yes	Yes	Yes	Yes	Yes	Yes
City effect	Yes	Yes	Yes	Yes	Yes	Yes	Yes	Yes	Yes
*F*	30.698	12.915	33.030	58.840	42.745	28.251	72.558	19.848	27.081
Hanse	0.180	0.813	0.766	0.134	0.170	0.614	0.159	0.069	0.235
AR(2)	0.051	0.151	0.150	0.602	0.064	0.930	0.640	0.675	0.877
Observations	3336	1668	1668	3336	1668	1668	3336	1668	1668

Notes: The *t*-statistics for the coefficients in parentheses ***, **, and * are for 1%, 5%, and 10% significance levels, respectively.

## References

[1] Liu Y., Wang S., Qiao Z., Wang Y., Ding Y., Miao C. (2019). Estimating the dynamic effects of socioeconomic development on industrial SO_2_ emissions in Chinese cities using a DPSIR causal framework. Resour. Conserv. Recycl..

[2] Wu J., Xiang W., Zhao J. (2014). Urban ecology in China: Historical developments and future directions. Landscape Urban. Plan..

[3] Zhu M., Shen L., Tam V.W., Liu Z., Shu T., Luo W. (2020). A load-carrier perspective examination on the change of ecological environment carrying capacity during urbanization process in China. Sci. Total Environ..

[4] Wang X. (2010). Urbanization path and city scale in China: An economic analysis. Econ. Res. J..

[5] Wang Z., Zhang X. (2019). The path selection for high quality development of China’s small towns in post-industrialization period. China Industr. Econ..

[6] The Economist (2018). China Is Trying to Turn Itself into a Country of 19 Super-Regionsm. https://www.economist.com/china/2018/06/23/china-is-trying-to-turn-itself-into-a-country-of-19-super-regions.

[7] World Business Council for Sustainable Development (WBCSD) (1996). Eco-efficient Leadership for Improved Economic and Environmental Performance.

[8] Wang Q., Su B., Zhou P., Chiu C. (2016). Measuring total-factor CO_2_ emission performance and technology gaps using a non-radial directional distance function: A modified approach. Energ. Econ..

[9] Shakouri R., Salahi M., Kordrostami S. (2019). Stochastic p-robust approach to two-stage network DEA model. Quantit. Fin. Econ..

[10] Battese G.E., Rao D.P., O’Donnell C.J. (2004). A metafrontier production function for estimation of technical efficiencies and technology gaps for firms operating under different technologies. J. Prod. Anal..

[11] Tone K. (2002). A slacks-based measure of super-efficiency in data envelopment analysis. Eur. J. Oper. Res..

[12] Pastor J.T., Lovell C.K. (2005). A global Malmquist productivity index. Econ. Lett..

[13] Wu G., Baležentis T., Sun C., Xu S. (2019). Source control or end-of-pipe control: Mitigating air pollution at the regional level from the perspective of the Total Factor Productivity change decomposition. Energ. Policy.

[14] Chung Y.H., Färe R., Grosskopf S. (1997). Productivity and undesirable outputs: A directional distance function approach. J. Environ. Manage..

[15] Han J., Gao M., Sun Y. (2019). Research on the measurement and path of urban agglomeration growth effect. Sustainability.

[16] Yuan Q. (2016). Do urban clusters promote the development of cities?. J. World Econ..

[17] Li P., Wang C., Zhang X. (2017). Did city cluster development help improve labor productivity in China?. J. Asia Pac. Econ..

[18] Ma J., Wang J., Szmedra P. (2019). Economic efficiency and its influencing factors on urban agglomeration—An analysis based on China’s top 10 urban agglomerations. Sustainability.

[19] Zheng S., Du R. (2020). How does urban agglomeration integration promote entrepreneurship in China? Evidence from regional human capital spillovers and market integration. Cities.

[20] Tao F., Zhang H., Hu Y., Duncan A.A. (2017). Growth of green total factor productivity and its determinants of cities in China: A spatial econometric approach. Emerg. Mark. Fin. Tr..

[21] Tang L., Li K. (2019). A comparative analysis on energy-saving and emissions-reduction performance of three urban agglomerations in China. J. Clean. Prod..

[22] Borck R., Pflüger M. (2018). Green cities? Urbanization, trade, and the environment. J. Reg. Sci..

[23] Xie H., Chen Q., Lu F., Wu Q., Wang W. (2018). Spatial-temporal disparities, saving potential and influential factors of industrial land use efficiency: A case study in urban agglomeration in the middle reaches of the Yangtze River. Land Use Policy.

[24] Song J., Feng Q., Wang X., Fu H., Jiang W., Chen B. (2019). Spatial association and effect evaluation of CO_2_ emission in the Chengdu-Chongqing urban agglomeration: Quantitative evidence from social network analysis. Sustainability.

[25] Huang Y., Li L., Yu Y. (2018). Do urban agglomerations outperform non-agglomerations? A new perspective on exploring the eco-efficiency of Yangtze River Economic Belt in China. J. Clean. Prod..

[26] Huang Y., Li L., Yu Y. (2018). Does urban cluster promote the increase of urban eco-efficiency? Evidence from Chinese cities. J. Clean. Prod..

[27] Bai Y., Deng X., Jiang S., Zhang Q., Wang Z. (2018). Exploring the relationship between urbanization and urban eco-efficiency: Evidence from prefecture-level cities in China. J. Clean. Prod..

[28] Cheng Y., Shao T., Lai H., Shen M., Li Y. (2019). Total-factor eco-efficiency and its influencing factors in the Yangtze River delta urban agglomeration, China. Int. J. Environ. Res. Pub. Health.

[29] Beltrán-Esteve M., Gómez-Limón J.A., Picazo-Tadeo A.J., Reig-Martínez E. (2014). A metafrontier directional distance function approach to assessing eco-efficiency. J. Prod. Anal..

[30] Yu Y., Peng C., Li Y. (2018). Do neighboring prefectures matter in promoting eco-efficiency? Empirical evidence from China. Technol. Forecast. Soc..

[31] O’Donnell C.J., Rao D.S.P., Battese G.E. (2008). Metafrontier frameworks for the study of firm-level efficiencies and technology ratios. Empir. Econ..

[32] Zhang N., Choi Y. (2013). Total-factor carbon emission performance of fossil fuel power plants in China: A metafrontier non-radial Malmquist index analysis. Energ. Econ..

[33] Zhou P., Ang B.W., Wang H. (2012). Energy and CO_2_ emission performance in electricity generation: A non-radial directional distance function approach. Eur. J. Oper. Res..

[34] Wursthorn S., Poganietz W., Schebek L. (2011). Economic-environmental monitoring indicators for European countries: A disaggregated sector-based approach for monitoring eco-efficiency. Ecol. Econ..

[35] Li W., Winter M., Kara S., Herrmann C. (2012). Eco-efficiency of manufacturing processes: A grinding case. CIRP Ann.-Manuf. Technol..

[36] Adjei Kwakwa P., Alhassan H., Aboagye S. (2018). Environmental Kuznets curve hypothesis in a financial development and natural resource extraction context: Evidence from Tunisia. Quantit. Fin. Econ..

[37] Zhu Y., Xia Y. (2019). Industrial agglomeration and environmental pollution: Evidence from China under New Urbanization. Energ. Environ.-UK.

[38] Krugman P. (1991). Increasing Return and Economic Geography. J. Polit. Econ..

[39] Glaeser E.L., Kahn M.E. (2010). The greenness of cities: Carbon dioxide emissions and urban development. J. Urban. Econ..

[40] Boix R., Trullén J. (2007). Knowledge, networks of cities and growth in regional urban systems. Pap. Reg. Sci..

[41] Fang C., Zhou C., Gu C., Chen L., Li S. (2017). A proposal for the theoretical analysis of the interactive coupled effects between urbanization and the eco-environment in mega-urban agglomerations. J. Geogr. Sci..

[42] Grossman G.M., Krueger A.B. (1995). Economic growth and the environment. Q. J. Econ..

[43] Brakman S., van Marrewijk C. (2013). Reflections on cluster policies. Cambridge J. Reg. Econ. Soc..

[44] Li Z., Liao G., Albitar K. (2019). Does corporate environmental responsibility engagement affect firm value? The mediating role of corporate innovation. Bus. Strategy Environ..

[45] Liu S., Zhu Y., Du K. (2017). The impact of industrial agglomeration on industrial pollutant emission: Evidence from China under New Normal. Clean. Technol. Environ..

[46] Verhoef E.T., Nijkamp P. (2002). Externalities in urban sustainability: Environmental versus localization-type agglomeration externalities in a general spatial equilibrium model of a single-sector monocentric industrial city. Ecol. Econ..

[47] Zhang K., Wang D. (2014). The Interaction and Spatial Spillover between Agglomeration and Pollution. China Industr. Econ..

[48] Lu M., Feng H. (2014). Agglomeration and emission reduction: An empirical study on the impact of urban size gap on industrial pollution intensity. J. World Econ..

[49] Glaeser E., Goodman J.C. (2011). Triumph of the city: How our greatest invention makes us richer, smarter, greener, healthier, and happier. Bus. Econ..

[50] Van der Panne G. (2004). Agglomeration externalities: Marshall versus Jacobs. J. Evol. Econ..

[51] Brandt L., Rawski T.G. (2008). China’s Great Economic Transformation.

[52] Wan Q., Zeng J.-X. (2013). The optimization of industrial structure of urban agglomeration from the perspective of spatial interaction: Case study of Wuhan Urban Agglomeration. Econ. Geogr..

[53] Jalil A., Feridun M. (2011). The impact of growth, energy and financial development on the environment in China: A cointegration analysis. Energ. Econ..

[54] Antonietti R., Cainelli G. (2011). The role of spatial agglomeration in a structural model of innovation, productivity and export: A firm-level analysis. Ann. Reg. Sci..

[55] Speldekamp D., Knoben J., Saka-Helmhout A. (2020). Clusters and firm-level innovation: A configurational analysis of agglomeration, network and institutional advantages in European aerospace. Res. Policy.

[56] Biggiero L., Sammarra A. (2010). Does geographical proximity enhance knowledge exchange? The case of the aerospace industrial cluster of Centre Italy. Int. J. Technol. Trans. Commercializ..

[57] Breschi S., Lenzi C. (2016). Co-invention networks and inventive productivity in US cities. J. Urban. Econ..

[58] Jacobs W., Koster H.R.A., van Oort F. (2013). Co-agglomeration of knowledge-intensive business services and multinational enterprises. J. Econ. Geogr..

[59] Yu X., Wu Z., Zheng H., Li M., Tan T. (2020). How urban agglomeration improve the emission efficiency? A spatial econometric analysis of the Yangtze River Delta urban agglomeration in China. J. Environ. Manag..

[60] Bertinelli L., Black D. (2004). Urbanization and growth. J. Urban. Econ..

[61] Timmer M.P., Los B. (2005). Localized innovation and productivity growth in Asia: An intertemporal DEA approach. J. Prod. Anal..

[62] Wang Q., Zhang C., Cai W. (2017). Factor substitution and energy productivity fluctuation in China: A parametric decomposition analysis. Energ. Policy.

[63] Lin B., Du K. (2013). The energy effect of factor market distortion in China. Econ. Res. J..

[64] Li J., Lin B. (2016). Green economy performance and green productivity growth in China’s cities: Measures and policy implication. Sustainability.

[65] Edwards J.R., Lambert L.S. (2007). Methods for integrating moderation and mediation: A general analytical framework using moderated path analysis. Psychol. Meth..

[66] Young A. (2002). Gold into base metals: Productivity growth in the People’s Republic of China during the reform period. J. Polit. Econ..

[67] Xiang J. (2011). The Estimation of Chinese Cities’ Fixed Capital Stock. Master’s Thesis.

[68] Portnov B., Erell E., Bivand R., Nilsen A. (2000). Investigating the effect of clustering of the urban field on sustainable growth of centrally located and peripheral towns. Int. J. Popul. Geogr..

[69] Portnov B.A., Schwartz M. (2009). Urban clusters as growth foci. J. Reg. Sci..

[70] Danison E.F. (1967). Why growth rates differ: Post-war experience in nine western countries. Wash. Brook. Inst..

[71] Yu B. (2015). Economic growth effects of industrial restructuring and productivity Improvement—Analysis of dynamic spatial panel model with Chinese City Data. China Industr. Econ..

[72] Dean J., Lovely M. (2010). China’s Growing Role in World Trade.

[73] Cole M.A. (2004). Trade, the pollution haven hypothesis and the environmental Kuznets curve: Examining the linkages. Ecol. Econ..

[74] Shahbaz M., Nasreen S., Abbas F., Anis O. (2015). Does foreign direct investment impede environmental quality in high, middle and low-income countries?. Energ. Econ..

[75] Chen Z., Kahn M.E., Liu Y., Wang Z. (2018). The consequences of spatially differentiated water pollution regulation in China. J. Environ. Econ. Manag..

[76] Sadorsky P. (2013). Do urbanization and industrialization affect energy intensity in developing countries?. Energ. Econ..

[77] Chen S., Golley J. (2014). “green” productivity growth in China’s industrial economy. Energ. Econ..

[78] Arellano M., Bover O. (1995). Another look at the instrumental variable estimation of error-components models. J. Econom..

